# A novel in-bed body posture monitoring for decubitus ulcer prevention using body pressure distribution mapping

**DOI:** 10.1186/s12938-024-01227-x

**Published:** 2024-03-15

**Authors:** Lindsay Stern, Geoff Fernie, Atena Roshan Fekr

**Affiliations:** 1 Institute of Biomedical Engineering, University of Toronto, 27 King’s College Cir, Toronto, Ontario M5S 3G9 Canada; 2 KITE Research Institute, Toronto Rehabilitation Institute, 550 University Ave, Toronto, Ontario M5G 2A2 Canada

**Keywords:** Decubitus ulcer, Sleeping postures, Convolutional neural network, Deep learning

## Abstract

**Background:**

Decubitus ulcers are prevalent among the aging population due to a gradual decline in their overall health, such as nutrition, mental health, and mobility, resulting in injury to the skin and tissue. The most common technique to prevent these ulcers is through frequent repositioning to redistribute body pressures. Therefore, the main goal of this study is to facilitate the timely repositioning of patients through the use of a pressure mat to identify in-bed postures in various sleep environments. Pressure data were collected from 10 healthy participants lying down on a pressure mat in 19 various in-bed postures, correlating to the supine, prone, right-side, and left-side classes. In addition, pressure data were collected from participants sitting at the edge of the bed as well as an empty bed. Each participant was asked to lie in these 19 postures in three distinct testing environments: a hospital bed, a home bed, and a home bed with a foam mattress topper. To categorize each posture into its respective class, the pre-trained 2D ResNet-18 CNN and the pre-trained Inflated 3D CNN algorithms were trained and validated using image and video pressure mapped data, respectively.

**Results:**

The ResNet-18 and Inflated 3D CNN algorithms were validated using leave-one-subject-out (LOSO) and leave-one-environment-out (LOEO) cross-validation techniques. LOSO provided an average accuracy of 92.07% ± 5.72% and 82.22% ± 8.50%, for the ResNet-18 and Inflated 3D CNN algorithms, respectively. Contrastingly, LOEO provided a reduced average accuracy of 85.37% ± 14.38% and 77.79% ± 9.76%, for the ResNet-18 and Inflated 3D CNN algorithms, respectively.

**Conclusion:**

These pilot results indicate that the proposed algorithms can accurately distinguish between in-bed postures, on unseen participant data as well as unseen mattress environment data. The proposed algorithms can establish the basis of a decubitus ulcer prevention platform that can be applied to various sleeping environments. To the best of our knowledge, the impact of mattress stiffness has not been considered in previous studies regarding in-bed posture monitoring.

## Introduction

Increasing age has become a predictor of decubitus ulcer formation due to the gradual decline of nutritional health, decreased mobility, and changes to the characteristics of the skin, such as decreased elasticity [1]. These ulcers can begin to develop within minutes due to prolonged applied body-weight forces, commonly occurring when one remains stationary in a seated or lying position [2]. This prolonged applied force, particularly to the bony regions of the body, can create cellular membrane breakage, ultimately resulting in cell death. This can initiate a cyclical process of cell death, inflammation, and ischemia, resulting in injury to the tissue [2]. It is also important to note that superficial skin injuries, typically caused by shear force and moisture, can indicate that damage to the tissue has already begun [2]. As of 2022, approximately 60,000 people die worldwide each year due to complications of decubitus ulcers [3]. It was also reported that those with such ulcers are 4.5 times more at risk of death than those who have similar health conditions with no decubitus ulcers [3]. In Canada, approximately 26% of patients across all healthcare settings suffer from decubitus ulcers; with as low as 15.1% of patients in community care facilities and as a high as 29.9% of patients in non-acute care settings [4]. Furthermore, 15% of elderly patients will develop decubitus ulcers within the first week of stay in the hospital, and within the first 4 weeks of stay in a long-term care facility [1].

The most common method to manage decubitus ulcers is through prevention, where the patient is required to reposition their body every two hours [2]. Unfortunately, in most cases this responsibility falls on caregivers and hospital staff, which can be a laborious task [5]. In addition, there is evidence to suggest that in clinical settings, many caregivers do not adhere to this repositioning program, suggesting that this prevention technique is not implemented properly [6]. Therefore, to alleviate some of the stress on the healthcare system and its workers as well as to ensure patients are receiving proper care, an in-bed posture and pressure monitoring systems need to be developed.

Currently, devices such as video infrared cameras or wearable technologies have been used to monitor in-bed postures and the corresponding duration of each posture. Nevertheless, these devices do have certain limitations. Video infrared cameras can be susceptible to environmental changes, such as the movement of a blanket, and have privacy concerns [5]. Wearable technologies, such as rings and wristbands, can obstruct sleep, reducing sleep quality, and are sensitive to motion artifacts [7]. Therefore, there is a need for an unobtrusive, privacy-preserving, and contactless system to monitor patients’ bodies during sleep and prompt caregivers to reposition them if necessary. The use of smart mats has become an area of investigation to monitor people in and outside of the hospital to reduce the work of caregivers, eliminate privacy concerns, and increase the accuracy of in-bed posture detection.

There are a variety of studies that have investigated in-bed posture detection using smart mats composed of either pressure or force sensors. Most of these studies either examine their subjects in a simulated environment, where the investigator instructs the subject to lie in different positions, or in a clinical environment, where the subjects can lie in any positions and is typically completed overnight. Stern et al. used an open access dataset with data collected in a simulated study with 13 participants lying in three in-bed posture classes (supine, right, and left). In this study, a mat with 2048 pressure sensors was placed on the bed and a 2D Convolutional Neural Network (CNN) was applied to classify each position. This study obtained an accuracy of 99.97% ± 0.03% and 99.62% ± 0.37% for fivefold and leave-one-subject-out (LOSO) cross-validation, respectively [8]. Similarly, Ostadabbas et al. conducted a simulated study with nine subjects lying in the same three in-bed posture classes. This study collected data from a mat containing 1,728 resistive sensors which was used to train a k-nearest neighbor algorithm. They achieved an accuracy of 98.4% using a holdout cross-validation technique [9]. Pouyan et al. conducted a similar simulated study with 13 participants, consisting of the same three in-bed posture categories. This study collected data from a pressure mat with 1,048 sensors to train a deep learning network to classify the postures, resulting in an accuracy of 82.70% while using a tenfold cross-validation technique [10]. These three studies mentioned above either grouped the supine and prone positions into a single class or neglected to collect data in the prone position, which creates an unrealistic analysis, as the prone posture is one of the four main sleeping categories. This approach undoubtedly leads to high accuracies as distinguishing between these two postures is challenging, due to similar positioning. Comparatively, Tang et al. conducted a simulated study with one participant lying in four in-bed posture categories (supine, prone, right, and left). This study used a force sensitive resistor mat with 171 sensors to collect in-bed posture data and trained the Inception-v3 CNN, achieving an accuracy of 87.0% using a holdout cross-validation technique [11]. Similarly, Matar et al. conducted a simulated study on 12 healthy participants lying in the same four in-bed posture classes on a piezo-resistive pressure mat containing 1,728 sensors. Using a feed-forward artificial neural network, this study achieved an accuracy of 97.9% using a holdout cross-validation technique with a nested LOSO cross-validation on the training set [12]. Although the majority of studies that include the prone position obtain lower accuracies than those that exclude this position, it is important to consider the prone position as that is a common sleeping posture. In addition, it is important to note that all these studies, except for the one conducted by Stern et al., did not validate their algorithms with leave-one-subject-out cross-validation, which is an essential technique to consider, as the goal of these algorithms will be to recognize unseen patient data. Furthermore, all these studies solely assessed their classification algorithms on a single type of mattress. To ensure that these algorithms can be applicable to a variety of sleeping environments, it is important to assess the performance of the algorithms on different mattress types with various associated stiffness.

In this study, we collect pressure data of in-bed postures from 10 healthy participants in three sleeping environments. We use pre-trained 2D and 3D CNN algorithms, referred to as the ResNet-18 and the Inflated 3D (I3D) classifiers, to classify the collected pressure distribution frames and videos into six distinct in-bed positions, including four main body postures (supine, prone, left, and right), an empty bed, and a seated position at the edge of the bed. We also evaluate how these algorithms perform on mattresses with various stiffness to ensure the performance of the pressure mat and algorithms are relatively consistent. We evaluate our algorithms using leave-one-subject-out (LOSO) and leave-one-environment-out (LOEO) cross-validation techniques, after addressing the class imbalance to compensate for fewer data points in the minority class.

## Results

The classification macro metrics, such as accuracy, sensitivity, specificity, and F1-score, shown in Equations  1 - 3, were calculated for each model to demonstrate the performance of the I3D and ResNet-18 algorithms.

1 $$\begin{aligned}{} & {} Accuracy = \frac{T_p + T_n}{T_p + T_n + F_p + F_n}\;\;\;\;\;\;\;\;\;\; Sensitivity = \frac{T_p}{T_p + F_n} \end{aligned}$$

2 $$\begin{aligned}{} & {} Specificity = \frac{T_n}{ T_n + F_p}\;\;\;\;\;\;\;\;\;\;Percision = \frac{T_p}{T_p + F_p} \end{aligned}$$

3 $$\begin{aligned}{} & {} F1-Score = 2*\frac{Percision * Sensitivity}{Percision + Sensitivity}, \end{aligned}$$

where T_P_, T_N_, F_P_, and F_N_ denote true positives, true negatives, false positives, and false negatives, respectively. Table  1 displays the performance of both the I3D and ResNet-18 CNNs with LOSO and LOEO cross-validations. As indicated in this table, the 2D CNN model consistently demonstrated the highest accuracies, achieving 92.07% ± 5.72% for LOSO and 85.37% ± 13.48% for LOEO. Likewise, all other performance metrics displayed in Table  1 provide similar trends. Therefore, it can be concluded that the ResNet-18 is a better model for classifying in-bed postures compared to the I3D model. This could be because the pressure distribution videos do not provide additional information, as the body remains stationary for the entire 16 s, making the temporal features of the 3D model less helpful for the classification. Furthermore, compared to the 2D approach that uses all frames individually, using the 3D approach involves combining frames into video files, leading to a reduction in size of the training data.

**Table 1:** Performance metrics for the I3D and ResNet-18 CNN models

	I3D model		ResNet-18 model	
	LOSO model	LOEO model	LOSO model	LOEO model
Accuracy (%)	82.22 ± 8.50	77.78 ± 9.76	92.07 ± 5.72	85.37 ± 14.38
F1-Score (%)	84.63 ± 6.75	80.75 ± 8.82	93.26 ± 4.14	87.40 ± 10.54
Sensitivity (%)	84.28 ± 6.79	79.56 ± 11.29	93.33 ± 4.81	87.69 ± 11.47
Specificity (%)	96.30 ± 1.57	95.36 ± 2.46	98.36 ± 1.15	97.01 ± 2.87

**Fig. 1:**
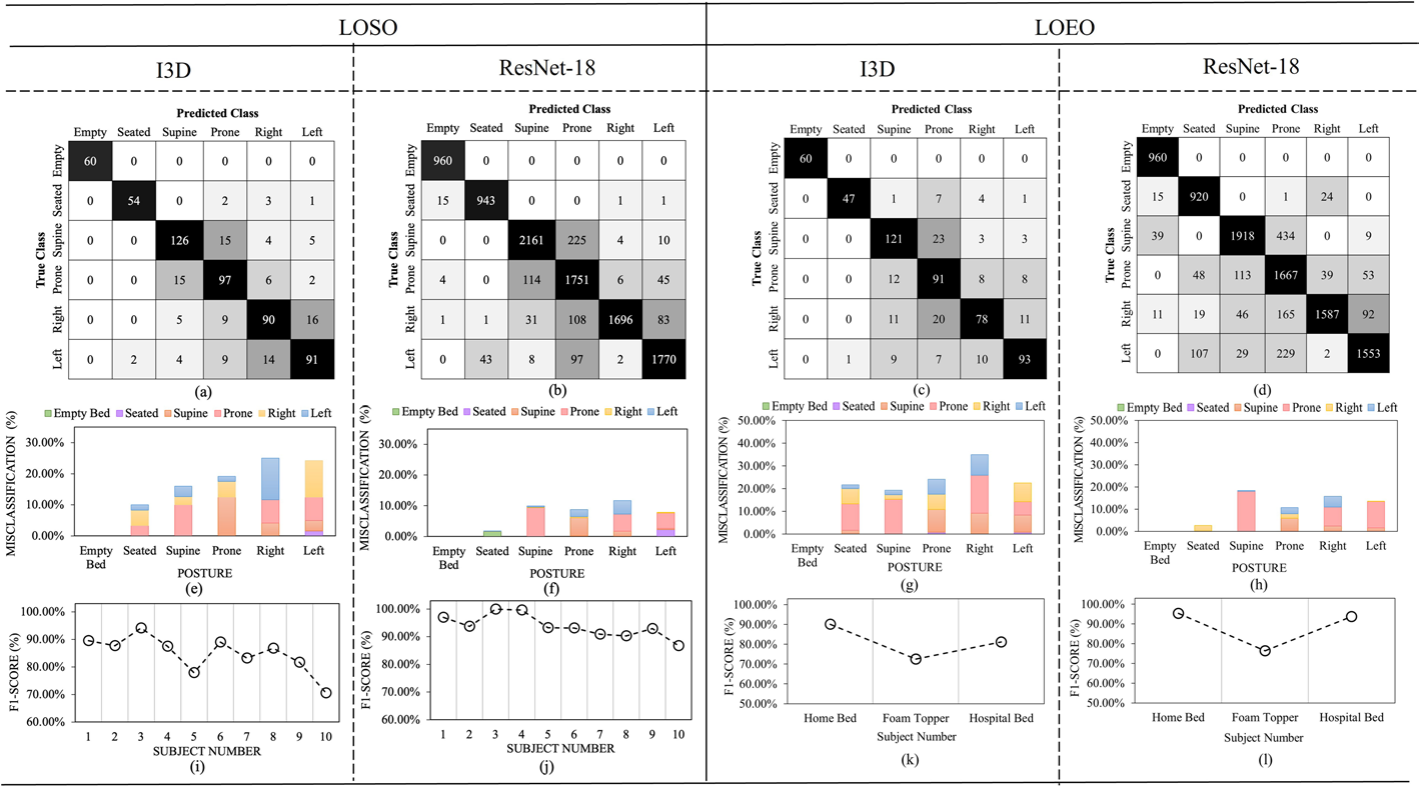
Performance visualizations for the LOSO and LOEO cross-validations of the I3D and ResNet-18 models. I3D model LOSO—**a** Confusion matrix, **e** misclassification rates plot, and **i** F1-scores pertaining to each subject. ResNet-18 model LOSO—**b** Confusion matrix, **f** misclassification rates plot, and **j** F1-scores pertaining to each subject. I3D model LOEO—(**c**) Confusion matrix, **g** misclassification rates plot, and **k** F1-scores pertaining to each subject. ResNet-18 model LOEO—**d** Confusion matrix, **h** misclassification rates plot, and **l** F1-scores pertaining to each subject

The left side of Fig.  1 displays the results for LOSO cross-validation and the right side shows the results for LOEO cross-validation. This figure shows that the empty bed class never got misclassified with either the I3D or the ResNet-18 models in both LOSO and LOEO cross-validations. Similarly, the seated class rarely became misclassified. For all models in LOSO and LOEO cross-validations, the prone (pink color in Fig.  1e–h) and supine (orange color in Fig.  1e–h) classes always got misclassified with each other, which was expected as these two postures have similar pressure distributions across the body. The right (yellow color in Fig.  1e) and left (blue color in Fig.  1e) classes primarily became misclassified with each other when using the I3D CNN model regarding the LOSO cross-validation, however when applying the ResNet-18 model, these two classes primarily became misclassified with the prone class. This misclassification of the right and left classes with the prone class can be seen throughout both the I3D and ResNet-18 models within LOEO cross-validation, shown in Fig.  1g, h.

Finally, it was observed that both the I3D and ResNet-18 models follow similar trends for the F1-scores for each participant, shown in Figure  1i, j. For example, subject 3 achieved the highest F1-score of 94.05% and 99.92% for the I3D and ResNet-18 models, respectively. Contrastingly, subject 10 received the lowest F1-score of 68.69% and 83.80% for the I3D and ResNet-18 models, respectively. This trend was also similar considering different types of mattresses in the LOEO cross-validation shown in  Fig. 1k, l. The home bed environment achieved the highest scores in both the I3D and ResNet-18 models, with 90.14% and 95.37%, respectively. Contrastingly, the home bed with a foam mattress topper achieved the lowest F1-score in both I3D and ResNet-18 models, with F1-scores of 68.10% and 76.35%, respectively.

## Discussion

Overall, the ResNet-18 algorithm performed better for both the LOSO and LOEO cross-validation techniques. This could be due to a couple of reasons: First, since the ResNet-18 algorithm classified individual frames, this created a larger training dataset, allowing the model to have a greater amount of information to learn from. Contrastingly, the I3D algorithm was trained on a single video, containing a sequence of frames, creating a smaller training dataset, and thus less information to learn from. Second, when testing the ResNet-18 model, only certain frames were misclassified, not an entire sequence of frames. Whereas, when testing the I3D model, an entire sequence of frames from a single video would become misclassified.

**Fig. 2:**
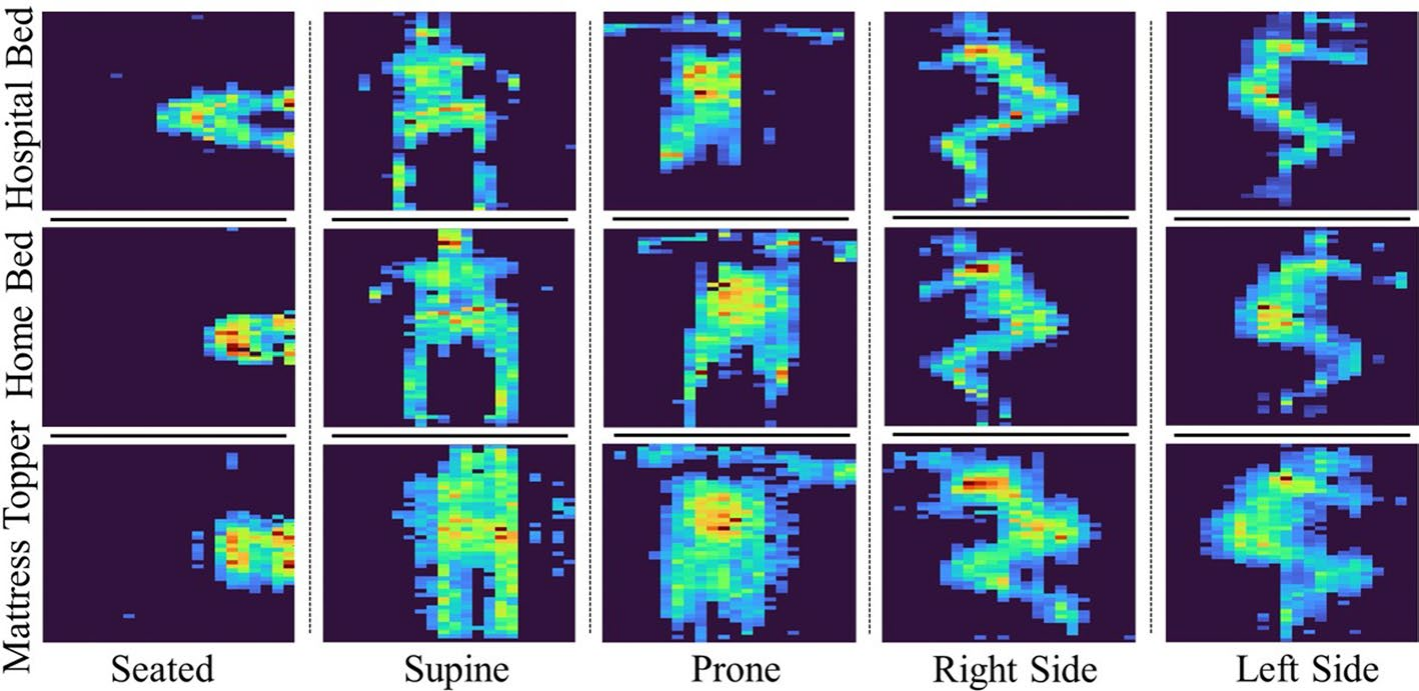
Frames of the four different sleeping postures and the seated position associated with the study environment (hospital bed, home bed, and home bed with the foam mattress topper)

In general, the LOSO cross-validation technique achieved higher values than the LOEO technique in both the I3D and ResNet-18 models. The LOSO cross-validation technique was trained on data from 9 subjects in all three study environments (home bed, home bed with foam mattress topper, and hospital bed) with leaving out 1 subject for testing. This was repeated 10 times. Comparatively, the LOEO cross-validation technique was trained with all subject data associated with two out of three mattress environments, leaving out the third mattress environment as the test set. Therefore, the algorithms consisted of more training data when utilizing the LOSO cross-validation technique compared to the LOEO cross-validation technique. Also, as shown in Fig.  2, the pressure distribution data vary in noise as the mattress stiffness level varies. The findings suggest that the home bed environment with a standard mattress (medium stiffness—S2) offers the best stiffness, as the pressure mat effectively captures the body with all limbs. On the other hand, the hospital bed’s stiffness (S1) posed some challenges regarding the pressure mat, particularly in capturing lighter body parts such as limbs, as observed in the prone position in Fig.  2. In contrast, the home bed equipped with a foam mattress topper (the least stiffness—S3) successfully captured the entire body, but introduced noise due to the either the softness of the foam topper or the placement of the pressure mat, which was in between the mattress and foam topper.

During the LOEO cross-validation technique, the instances where the models were trained on the hospital and home bed environments and tested on the home bed with the foam mattress topper, received the lowest performance metrics, bringing the overall performance of the I3D and ResNet-18 models down. This occurrence is likely due to the high levels of noise that was introduced in the test dataset, which the models did not see beforehand.

**Fig. 3:**
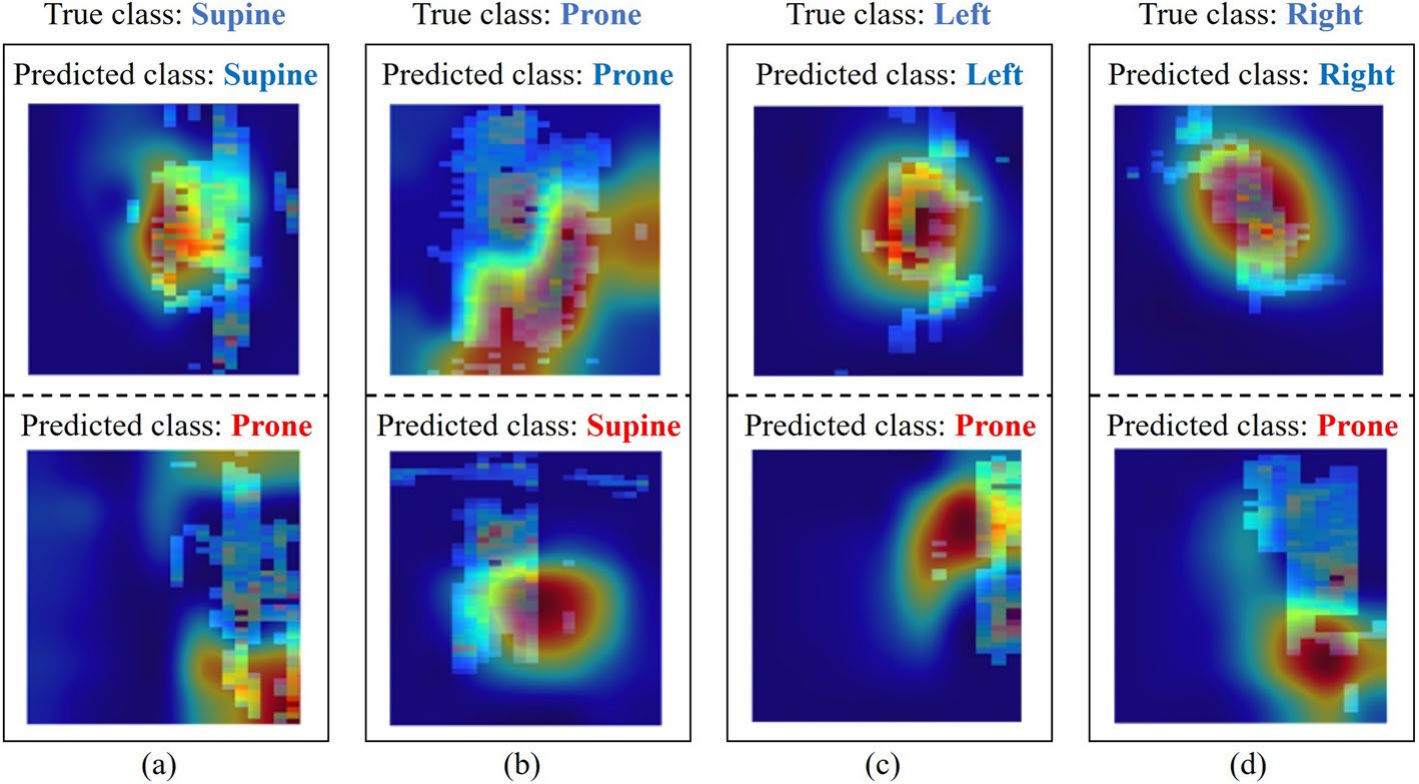
ResNet-18 Model—Grad-CAM frames for the correctly and incorrectly predicted images associated with the **a** supine,  **b** prone, **c** left, and **d** right classes

To further evaluate the performance of the ResNet-18 model, a Gradient weighted Class Activation Map (Grad-CAM) was used to highlight the important regions of the images for model prediction. Grad-CAM enhances our understanding of the regions that the models prioritize and focus on while classifying the images. Figure  3 displays examples of the Grad-CAM images for correctly and incorrectly classified images using the ResNet-18 algorithm. The more intense red colors represent the areas of the image that the model was more focused on and considered significant for the prediction. In the first example, in Fig.  3a, the subject was in the supine class, lying in the bent knee and the straight subclasses for the correctly and misclassified predictions, respectively. For the prone class, shown in Fig.  3b, the subject was lying in the straight and the arms crossed subclasses for the correctly and misclassified predictions, respectively. For the left and right classes, shown in Fig.  3c, d, the subject was lying in the log subclass and the bent knees behind subclass for both the correct and misclassified predictions, respectively. It was observed that the model primarily focuses on the torso area when classifying the body position correctly for the supine, left, and right classes. However, for the prone class, it was observed that the model focuses primarily on the limbs of the subject instead. Contrastingly, when the supine, left, and right classes were misclassified with the prone class, the model was looking at the limbs of the participants, whereas, when the prone class was misclassified as supine, the model was focusing on the torso of the subject instead.

Table  2 compares previous studies that have used smart mats to collect in-bed posture data and classify these data into at least four classes using various algorithms and cross-validation techniques. All the papers presented in Table  2 included the four main sleeping postures of supine, prone, left, and right side. However, the data within each paper were collected from different subjects, positions, and smart mats. These variables represent significant components that could potentially influence the performance of in-bed posture classification algorithms. Therefore, it is essential to consider these factors when comparing the outcomes of these studies. The majority of the papers did use a subject specific cross-validation method, aside from [11] which included only a single subject. However, none of these papers investigated the impact of different mattresses with various stiffness levels. Thus, to our knowledge this study is the first that evaluates the classification model not only by considering unseen subject data (LOSO) but also unseen mattress environment data (LOEO). It can be seen from our results that the introduction of unseen environments will decrease the performance of the algorithm.

**Table 2:** A comparison between previous literature and our proposed models (bolded values) with regard to the classification of a minimum of four in-bed postures, containing at least supine, prone, right, and left

Year & Refs.	# Subs	# Postures	Algorithm	Cross-Validation Method	Performance
2019 [12]	12	4	FFAN^1^	LOSO	ACC^2^: 97.9%
2020 [13]	7	4	kNN^3^	LOSO	ACC: 79.02%
2021 [11]	1	6	Inception-v3	Holdout	ACC: 90.5%
2022 [14]	6	4	SRC^4^	6-Fold & LOSO	ER^5^: 0.09
**Proposed Models**	**10**	**6**	**I3D DNN** ^6^	**LOSO**	**ACC: 82.22%**
				**LOEO**	**ACC: 77.78%**
			**ResNet-18**	**LOSO**	**ACC: 92.07%**
				**LOEO**	**ACC: 85.37%**

^1^FFAN: Feed-Forward Artificial Neural Network, ^2^ACC: Accuracy, ^3^kNN: k-Nearest Neighbor, ^4^SRC: Sparse Representation Classification, ^5^ER: Error Rate, ^6^DNN: Deep Neural Network

Although our algorithms achieved high scores for distinguishing among different body positions, it is important to recognize some limitations. First, the data collected in this experiment were collected on healthy participants. Therefore, in the future we will replicate the population group that is most likely to use a pressure mat for pressure injury monitoring. In addition, although there was an attempt to create variation in our dataset with various sleeping postures relating to each class, it should be recognized that the data were collected during the daytime for 1–2 min with the participants lying in specific instructed positions. In the future, it would be more beneficial to collect data in an overnight study, where the participants can lie in their normal sleeping postures, to train a model on more realistic positions. In the future we will also consider analyzing the different mattress environments separately to determine how the various stiffness may affect the training and testing of the model, which will also create more targeted classifiers depending on mattress stiffness. Another limitation of our study arises from the different placements of the pressure mat in various sleeping environments. In the cases of the “hospital bed” and “home bed” mattresses, we positioned the pressure mat directly on top of the mattress, with participants lying on the mat directly. However, for the “foam mattress topper” environment, we placed the pressure mat between the mattress and the foam topper, not directly beneath the participant. This approach aimed to mimic the mat’s realistic use, as we wanted to avoid altering the pressure-alleviating characteristics of the foam topper. While this placement ensures practical relevance, it introduces a variable in the study’s setup that may affect the comparability of results across different sleeping environments. In the future, we will consider evaluating the placement of the pressure mat on top of the foam topper to better evaluate the effects of the low stiffness.

## Conclusion

An Inflated 3D model and the ResNet-18 2D pre-trained CNN were used to differentiate between the supine, prone, right, left side, empty bed, and seated classes. The LOSO and LOEO cross-validation methods were used to evaluate the models’ performances. In summary, the 2D classifier outperformed the 3D model, resulting in accuracies of 92.07% ± 5.72% and 85.37% ± 14.38% for LOSO and LOEO cross-validation techniques, respectively. This indicates that the spatial–temporal features of the video data did not offer any additional information for the 3D CNN model, considering the stationary position of the body. These results show that the 2D CNN algorithm will be able to classify the in-bed postures of unseen patients quite well on unseen mattress environments, the best performing model trained on the hospital bed and foam mattress topper and tested on the home bed environment. However, it is important to note that the performance of the model decreased as significant noise from the pressure distribution frames were introduced. Therefore, further pre-processing techniques may be required to help remove the noise introduced into the pressure distribution image due to softness and pressure mat placement. Eventually, these models can be used as a preventative measure for decubitus ulcers, to either notify caregivers when it is time to change the position of the patients or can be used to monitor overnight sleeping to determine how much a person is moving in their sleep, thus evaluating the sleep quality of the person.

## Methods

### Dataset

**Fig. 4:**
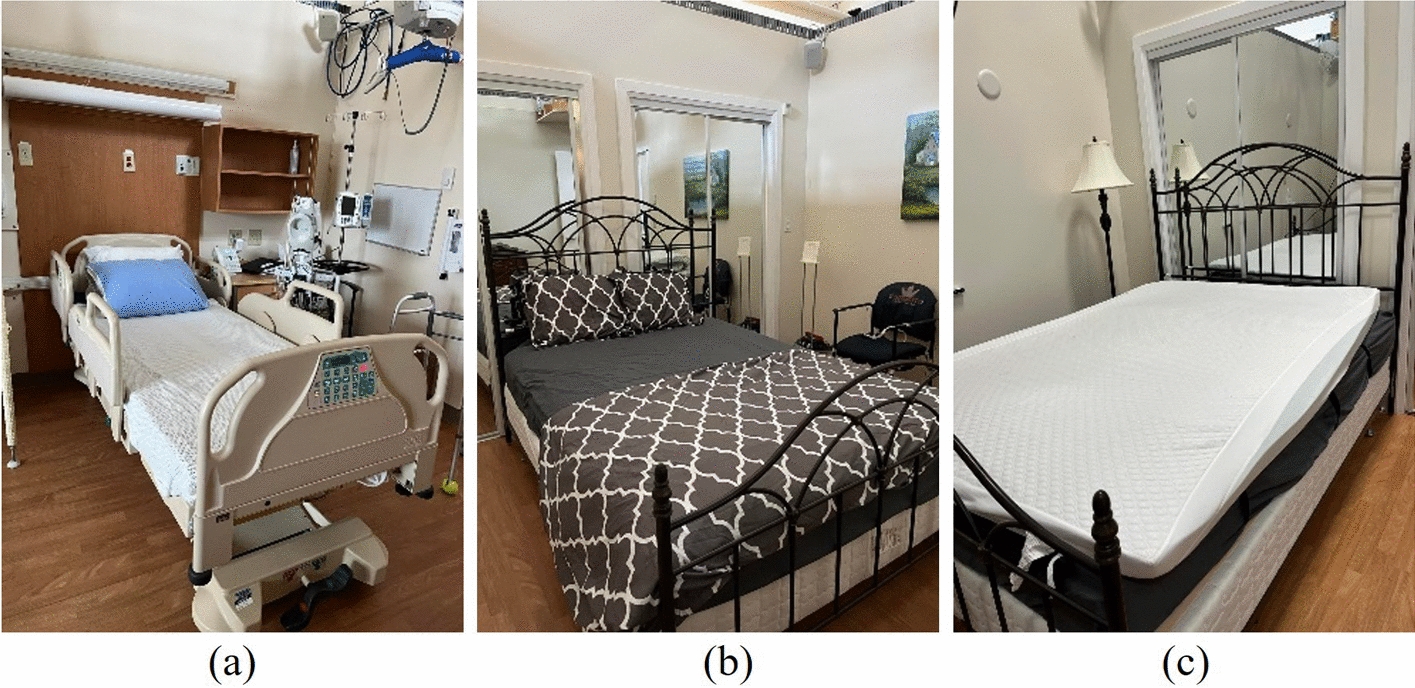
The three different environments evaluated: **a** hospital bed, **b** home bed, **c** home bed with foam mattress topper

The dataset used in this paper was collected using the SensingTex pressure mat composed of 1056 sensors with a sampling rate of 1Hz [15]. The SensingTex pressure mat was placed on three types of mattresses, shown in Fig.  4. Figure  4a displays a hospital bed that is relatively stiff (S1) compared to the other environments. Figure  4b shows a mattress that is typically found in homes (medium stiffness - S2). Last, Fig.  4c displays a foam mattress topper placed on top of the home bed, which creates a relatively soft environment (S3) compared to the other two mattresses. The SensingTex pressure mat was placed on top of the hospital and home bed, in Fig.  4a and b, and placed in between the mattress and foam mattress topper in Fig.  4c.

**Fig. 5:**
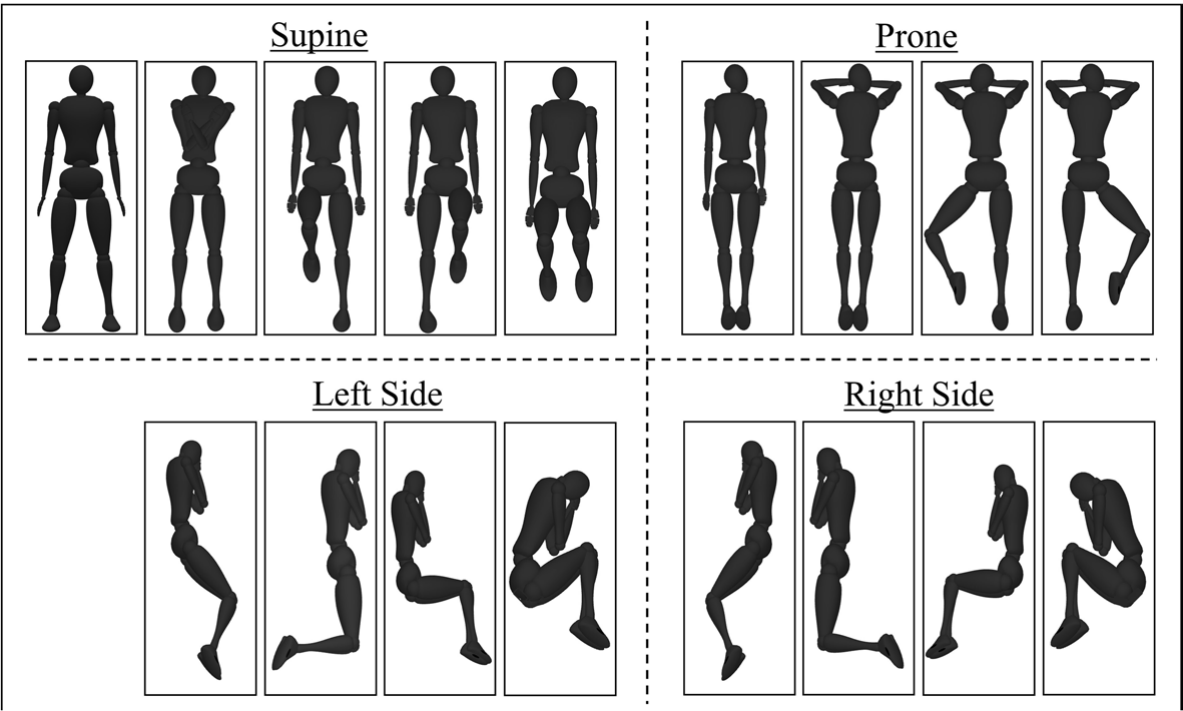
The 17 different in bed postures correlating to supine, prone, left side, and right side sleeping positions

Pressure data were collected from 10 healthy participants in all three of these environments. Each participant’s age, height, and weight were recorded, with ages ranging from 19- to 25-year old, heights from 161 cm to 183 cm, and weights from 55 kg to 95 kg. Participants were asked to lie down in 17 different postures for 1 min, shown in Fig.  5. These positions include 5 postures in the supine (straight, arms crossed, left knee bent, right knee bent, and both knees bent), 4 in the prone (straight, arms crossed, left knee bent, and right knee bent), 4 lying on the right side (log, bent knees behind, bent knees forward, and fetus), and 4 lying on the left side (log, bent knees behind, bent knees forward, and fetus). In addition, participants were asked to sit at the edge of each mattress when they entered and exited the bed and pressure data were also collected on empty beds. The data were collected under REB approval at the University Health Network in Toronto. Informed consents were signed by all participants prior to the data collection and they agreed to the anonymous publication of their data for future research.

### Data preprocessing

The pressure data were initially collected as numerical pressure values, where each row of data corresponded to a frame of pressure. The data were transformed into 22$\times$48 matrices to create pressure distribution images, which were subsequently combined into a single video file. Each video includes 16 frames (16 s videos) with a size of 112$\times$112 pixels for computational purposes.

### Inflated 3D model

**Fig. 6:**
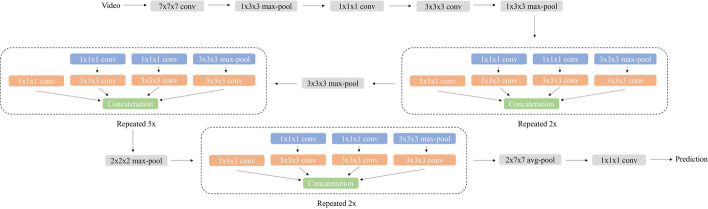
The Inflated Inception-V1 CNN architecture, adapted from [16]

The Inflated 3D (I3D) model is a pre-trained two-stream CNN video classifier created by Carreira and Zisserman [16]. This classifier has been trained on the Kinetics-400 dataset, which includes 400 human action (e.g. drawing, drinking, laughing, punching, etc.). Each class consists of 400–10 s videos, totaling to 240,000 training videos [16]. This classifier consists of the Inflated Inception-v1 3D CNN classifier which is trained twice: once on RGB data, and once on optical flow data, which was obtained from the TV-L1 (Total Variation - L1 norm) algorithm. The final prediction of the model is then averaged during validation [16]. The architecture of the Inflated Inception-v1 algorithm can be seen in Figure  6, which was adapted from [16].

Using transfer learning, we trained the I3D CNN algorithm on video data collected in this study, corresponding to each posture, resulting in a total of 630 videos: 60 videos in the empty bed class, 60 videos in the seated class, 150 videos in the supine class, 120 videos in the prone class, 120 videos in the right side class, and 120 videos in the left side class. Our trained I3D CNN was evaluated using two cross-validation techniques: leave-one-subject-out (LOSO) and leave-one-environment-out (LOEO). The LOSO cross-validation technique consists of training the algorithm using almost all subject data except for one subject, and then validating the trained algorithm on the unseen left-out subject data. For example, in this study, we trained the algorithm on data from nine subjects and then validated this algorithm on the tenth unseen subject data. We repeated this ten times to understand how the algorithm will perform on various unseen subject data. The LOEO cross-validation technique consists of training the algorithm on almost all mattress environments, except for one, and then validating this trained model on the unseen mattress environment. For example, in this study we trained the algorithm on the data from the hospital bed and the home bed environments and then validated this algorithm on the unseen data from the home bed with the foam mattress topper environment. We repeated this approach three times, training on two of the environments and then leaving the third environment out for validation purposes. These two techniques allowed us to understand the performance of the algorithm on unseen subject data and unseen mattress environment data.

### ResNet-18 model

**Fig. 7:**
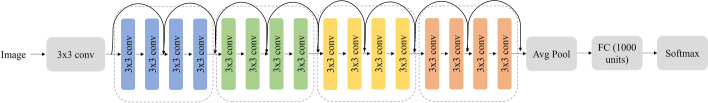
The ResNet-18 CNN architecture, adapted from [17]

A pre-trained 2D CNN model was used to compare the results with the I3D model. The ResNet-18 model is 18 layers deep and has previously been trained on the ImageNet database, which is composed of over a million of images pertaining on 1000 object categories [17]. The architecture of this algorithm can be seen in Fig.  7, which was adapted from [17]. Transfer learning was used to apply the architecture of the ResNet-18 model to the in-bed posture data collected in this study. We trained this 2D CNN on image data corresponding to the frames associated with each video used within the I3D model. This resulted in a total of 10,080 images: 960 images in the empty bed class, 960 images in the seated class, 2400 images in the supine class, 1920 images in the prone class, 1920 images in the right-side class, and 1920 images in the left-side class. Our trained 2D CNN was evaluated similarly to the I3D algorithm, using both LOSO and LOEO cross-validation techniques.

### Hyperparameter tuning and imbalanced dataset

The I3D and 2D CNN models were trained and validated using four main hyperparameters: batch size, number of iterations, number of epochs, and learning rate. These hyperparameters were tuned using a grid search technique with a fivefold cross-validation for both algorithms. This tuning process consisted of evaluating the performance of both models using a combination of various hyperparameters to determine the best combination. For both models, the search range for the number of iterations was between 100–500, 10–100 for epoch size, and 0.1$-$0.001 for learning rate. For the I3D model, the batch size search range was between 16–512. For the ResNet-18 model, the batch size search range was between 16 and 1204. The best hyperparameter combination for the I3D CNN consisted of a batch size of 39 with 300 iterations, epoch size of 28, and learning rate of 0.01. For the ResNet-18 CNN, the best determined hyperparameters were a batch size of 570, with 150 iterations, epoch size of 10, and a learning rate of 0.01.

In this study, the dataset collected was imbalanced, meaning that some classes had more data (images or videos) than others. The supine category had the most data collected, and the empty bed and seated categories had the least amount of data. To overcome this challenge, a ‘class weights’ technique was used. This method assigns a weight to each class so that the class with more samples will have a lower weight than the minority class, meaning that both CNN models can learn equally from all classes and will not be biased to the class with more samples. The weights for each class were determined using Equation  4.

4 $$weight\,\left( {class\left( x \right)} \right) = \frac{{total{\mkern 1mu} \,{\mkern 1mu} number{\mkern 1mu} \,{\mkern 1mu} of{\mkern 1mu} {\mkern 1mu} videos{\mkern 1mu} {\mkern 1mu} \,(or{\mkern 1mu} {\mkern 1mu} images)}}{{total{\mkern 1mu} {\mkern 1mu} \,number\,of\,classes*number{\mkern 1mu} \,of\,videos{\mkern 1mu} \,{\mkern 1mu} \left( {or{\mkern 1mu} {\mkern 1mu} images} \right){\mkern 1mu} {\mkern 1mu} \,in{\mkern 1mu} {\mkern 1mu} \,class\,x}}$$

## Data Availability

Not applicable.
